# Supplementary material for: Where to rewild? A conceptual framework to spatially optimize ecological function
Source: Proc Biol Sci. 2020 Mar 4;287(1922):20193017. doi: 10.1098/rspb.2019.3017 (PMC7126074; doi:10.1098/rspb.2019.3017)
Supplement: Appendix A: Submodels of the Guam Case Study;Appendix B: Supplementary Outputs and Figures [file rspb20193017supp1.docx]

Appendix A

*Function Score*

$$F_{i}=\frac{C_{d}}{T_{d}}*pD_{n}*P_{i}$$

with F_i_ being the function score of cell i, C_d_ being the number of intact forest cells within maximum dispersal distance by the Såli (500m, [66]) , T_d_ being the total number of cells within the same distance, pD_n_ being the probability of dispersal from the closest intact forest cell calculated using the Såli dispersal kernel (Log-normal distribution with a shape of 0.97 and a mean of 4.53) and P_i_ being the priority score of the land cover assigned to the cell I (1 for intact limestone forest, 0.8 for mixed introduced forest, and 0.6 for *Leucaena* thicket). The priority scores were decided based on how much complementary effort would be needed, in addition to seed dispersal, to restore landcovers to an intact state. Mixed introduced forest would also need ungulate control, scoring it lower than intact forest. *Leucaena* thicket would need vine control in addition to ungulate hunting and seed dispersal, scoring it lower than both other landcovers.

*Rewilding Score*

$$R_{i}=\sum_{j}^{T_{h}} (F_{j}*\frac{H-D_{j}}{H})$$

with T_h_ being the total number of cells within home-range, j being each cell within the potential home-range of the Såli if they were reintroduced in cell i, F_j_ being the function score of cell j, H being the maximum homerange distance (1083m), and D_j_ being the distance between cell i and cell j.

*Management Score*

$$M_{k}=\frac{\sum_{j}^{T_{k}} R_{j}}{T_{k}}$$

with T_k_ being the total number of cells within the management unit k and R_j_ being the rewilding score of each cell.

*Creating management units in ArcGIS*

We used the landcover map to separate the cells between both the developed and undeveloped categories. We then used the “spatially constrained multivariate clustering” function from the spatial statistics toolbox in ArcGIS to cluster cells from the same category into clusters, based on similarity of their rewilding score values, and cluster size. Developed clusters range from 1 to 100 hectares, while non-developed clusters ranged from 100 to 300 hectares.

Management units are then sub-divided along primary roads because it is not feasible to construct effective exclosure fences across these frequented roads. In addition, roads act as natural barriers to snake movement and provide easy access for trapping points.

Finally, we also applied a “minimum area selection” to management units by deleting those too small to be individually managed (less than 1 hectare) after being sub-divided by roads.

Appendix B

Table B.1: Summary of potential habitats identified for the Såli across the island

| Landcover | Percentage of Island Area | Percentage of Landcover which is Såli habitat |
| --- | --- | --- |
| Agriculture | 0.5% | 2% |
| Casuarina | 0.1% | 30% |
| Coastal Scrub | 0.4% | 54% |
| Coconut | 1% | 52% |
| Developed | 20% | 19% |
| Grassland | 17% | 4% |
| Leucaena Thicket | 4% | 12% |
| Mixed Grass/Herbaceous | 5% | 24% |
| Mixed Introduced Forest | 25% | 12% |
| Intact Forest | 10% | 98% |
| Scrub/Shrub | 8% | 30% |
| Total Percent of the island that is potential habitat for the Såli | | 22% |

Table B.2: Summary of the spatial statistics of the three prioritized forest landcovers that are assigned function scores.

| Landcover | Percentage of island area | Cells with a function score higher than 0 |
| --- | --- | --- |
| Intact Limestone Forest | 10% | 100% |
| Mixed Introduced Forest | 25% | 14% |
| *Leucaena* Thicket | 4% | 28% |
| Total percent of cells of the these 3 landcovers that would benefit from seed dispersal | | 37% |
| Total percent of the island which would benefit from seed dispersal | | 14.5% |


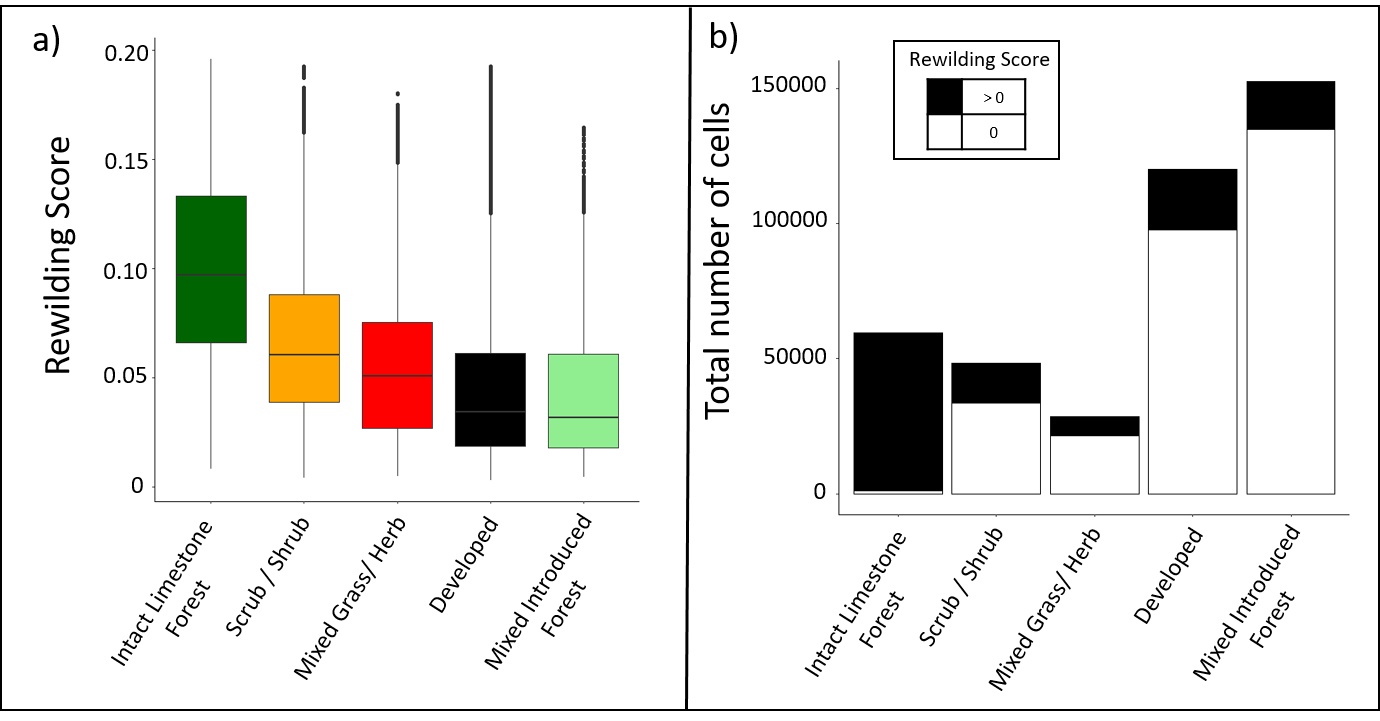


Figure B.1: Summary of the rewilding score for five common landcover types on Guam. a) Boxplots of rewilding score for positively scored cells of each landcover. b) Barplots of the distribution of rewilding scores for all cells of each landcover.


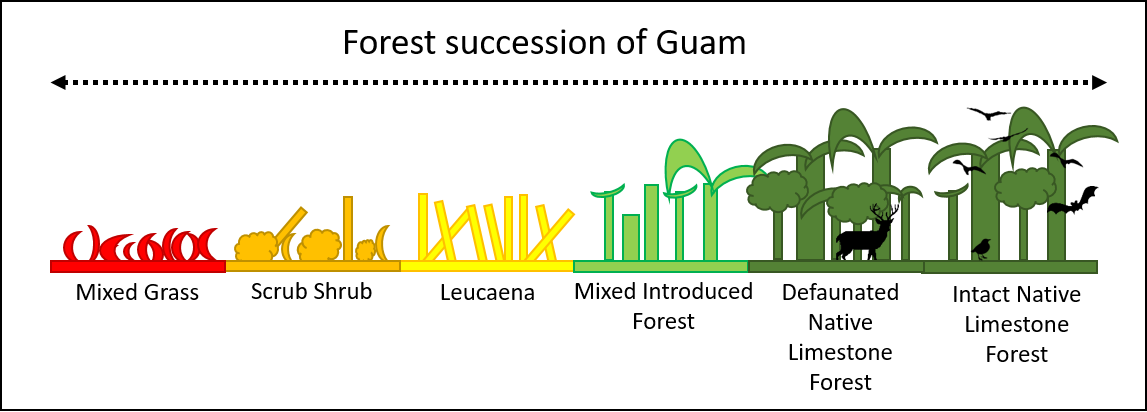


Figure B.2: Forest succession on the island of Guam. Rewilding the forests with seed dispersers and excluding invasive ungulates are obligatory steps to restore the intact forests throughout the island. In intact native forest, we find all native seed dispersers. In defaunated native forest, these dispersers are no longer present, and we find invasive ungulates.


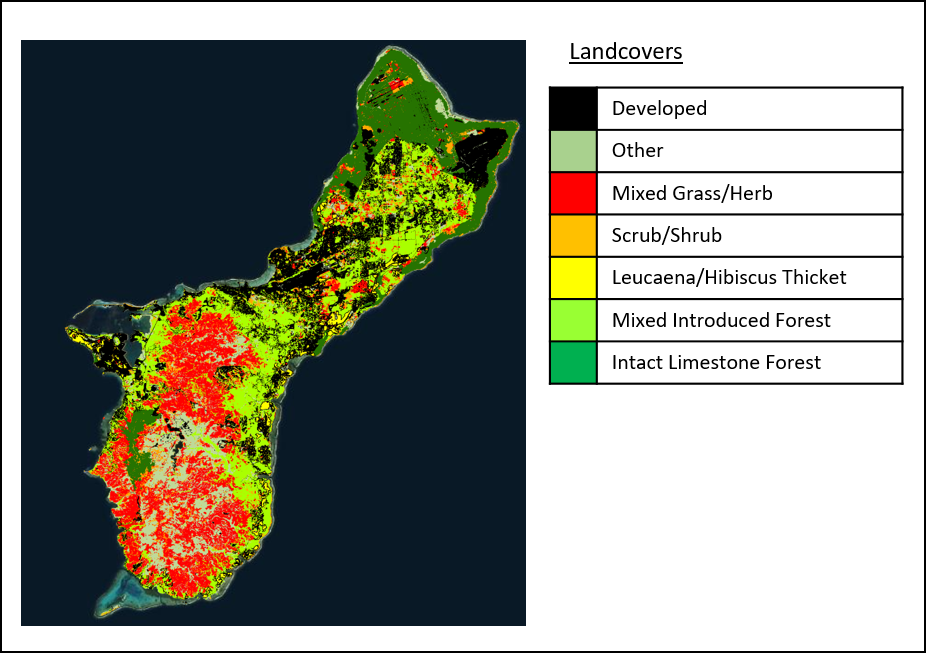


Figure B.3: Landcover map of Guam


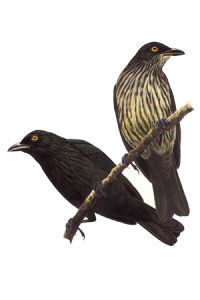

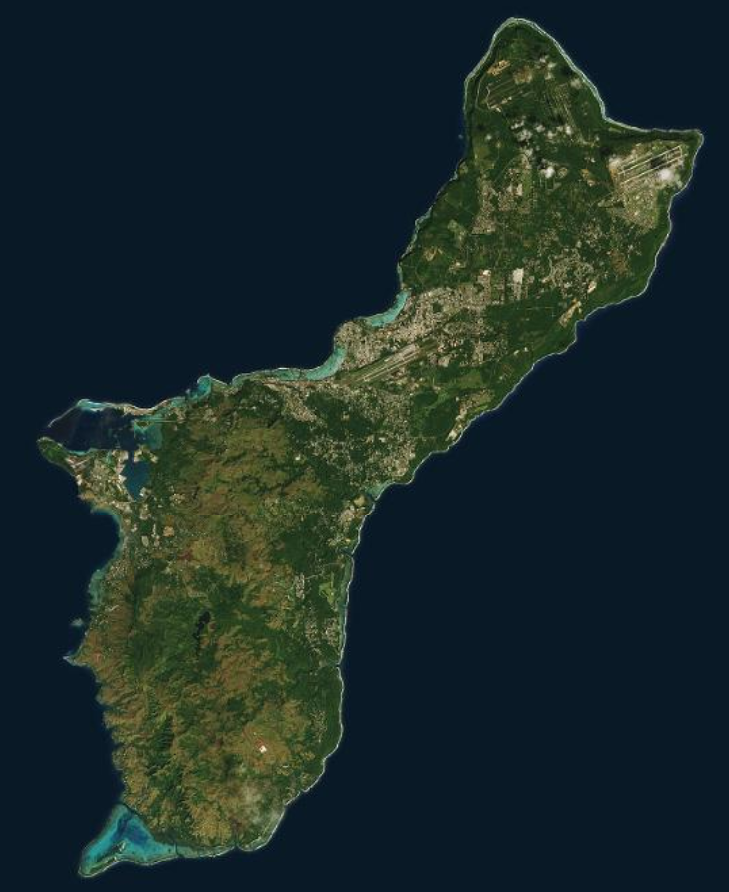

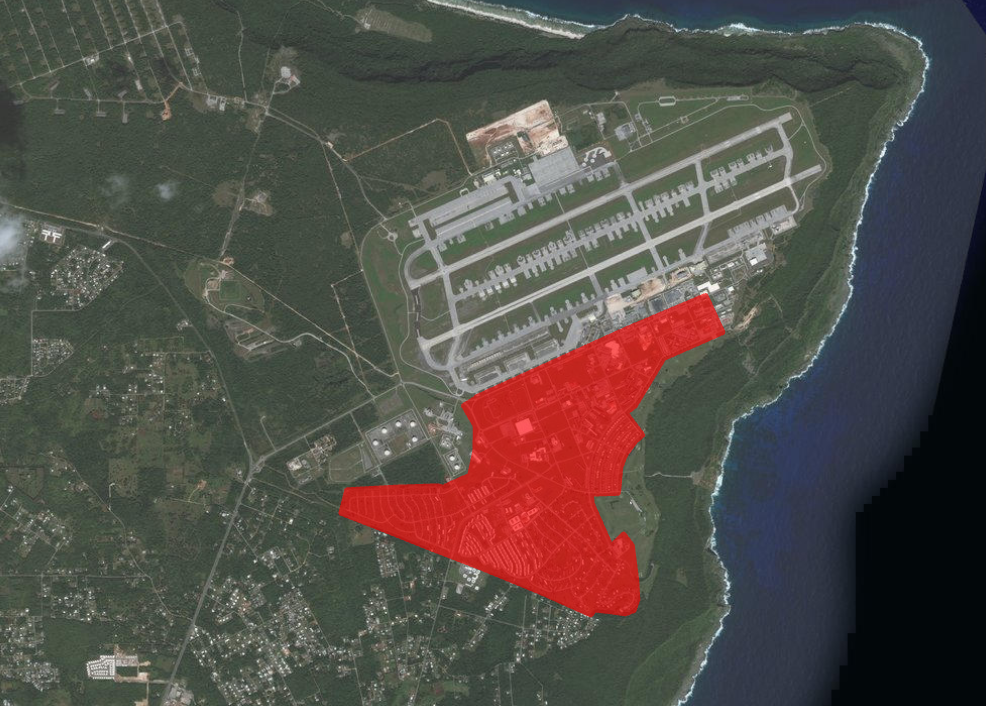


Figure B.4: Map of where the remnant population of Sali is found on Guam.
